# GWIPS-viz: development of a ribo-seq genome browser

**DOI:** 10.1093/nar/gkt1035

**Published:** 2013-10-31

**Authors:** Audrey M. Michel, Gearoid Fox, Anmol M. Kiran, Christof De Bo, Patrick B. F. O’Connor, Stephen M. Heaphy, James P. A. Mullan, Claire A. Donohue, Desmond G. Higgins, Pavel V. Baranov

**Affiliations:** ^1^School of Biochemistry and Cell Biology, University College Cork, Cork, Ireland, ^2^School of Medicine & Medical Science, Conway Institute, University College Dublin, Dublin 4, Ireland and ^3^Howest, University College West Flanders, Rijselstraat 5, 8200 Bruges, Belgium

## Abstract

We describe the development of GWIPS-viz (http://gwips.ucc.ie), an online genome browser for viewing ribosome profiling data. Ribosome profiling (ribo-seq) is a recently developed technique that provides genome-wide information on protein synthesis (GWIPS) *in vivo*. It is based on the deep sequencing of ribosome-protected messenger RNA (mRNA) fragments, which allows the ribosome density along all mRNA transcripts present in the cell to be quantified. Since its inception, ribo-seq has been carried out in a number of eukaryotic and prokaryotic organisms. Owing to the increasing interest in ribo-seq, there is a pertinent demand for a dedicated ribo-seq genome browser. GWIPS-viz is based on The University of California Santa Cruz (UCSC) Genome Browser. Ribo-seq tracks, coupled with mRNA-seq tracks, are currently available for several genomes: human, mouse, zebrafish, nematode, yeast, bacteria (*Escherichia coli* K12, *Bacillus subtilis*), human cytomegalovirus and bacteriophage lambda. Our objective is to continue incorporating published ribo-seq data sets so that the wider community can readily view ribosome profiling information from multiple studies without the need to carry out computational processing.

## INTRODUCTION

Ribosome profiling is based on the isolation of messenger RNA (mRNA) fragments protected by ribosomes followed by massively parallel sequencing of the protected fragments or footprints. This allows the measurement of ribosome density along all mRNA transcripts present in the cell providing genome-wide information on protein synthesis (GWIPS) *in vivo* (1). The ribosome profiling technique, also known as ribo-seq, was first carried out in *Saccharomyces cerevisiae* (2). Since the original publication, the technique has been carried out in many organisms including *Homo sapiens* (3–11) *Mus musculus* (3,7,9,12,13) *Danio rerio* (14), *Caenorhabditis elegans* (4,15), *S. cerevisiae* (16,17), *Escherichia coli* (18,19), *Bacillus subtilis* (19), human cytomegalovirus (20) and bacteriophage lambda (21).

To date, there have been two main strategies of ribosome profiling: ribosome profiling of initiating ribosomes and ribosome profiling of elongating ribosomes. For a review on the usages and advantages of each approach, please see (22).

The majority of published studies using ribosome profiling provide the raw sequencing data in NCBI’s Sequence Read Archive (SRA) (23). In addition, most published ribosome profiling experiments have corresponding naked mRNA controls, where total mRNA is randomly degraded to yield fragments of a size similar to ribosome protected fragments. For simplicity, here we refer to it as mRNA-seq. mRNA-seq is carried out under the same experimental conditions. It helps to take into account the differential abundance of mRNA between experimental conditions and to monitor technical biases associated with complementary DNA library generation and sequencing.

Owing to the increasing popularity of the ribo-seq technique, the number of ribosome profiling experiments is expected to increase dramatically in the near future. However, the visualization of ribosome profiling data in a browser first requires preprocessing and aligning the raw sequencing reads. As with any type of next-generation sequencing data, demands are placed on biomedical researchers in terms of time, data storage, computational knowledge and prototyping of computational pipelines (24). Web-based integrative framework tools such as Galaxy (25) provide centralized platforms for researchers to carry out next-generation sequencing alignment pipelines. However, because of decreasing costs, the coverage depth of ribo-seq and corresponding mRNA-seq data is continually increasing resulting in ever larger data sets. Consequently the computational resources required to process such data and the computer memory required to store such data may not be available to many biologists. The time required to download, preprocess and align the raw data may be the most limiting factor of all for time-poor researchers.

To address these issues, we introduce GWIPS-viz (http://gwips.ucc.ie), a free online browser that is pre-populated with published ribo-seq data. The aim of GWIPS-viz is to provide an intuitive graphical interface of translation in the genomes for which ribo-seq data are available. Users can readily view alignments from many of the published ribo-seq studies without the need to carry out any computational processing. GWIPS-viz is based on a customized version of the University of California Santa Cruz (UCSC) Genome Browser (http://genome.ucsc.edu) (26). Ribo-seq tracks, coupled with mRNA-seq tracks, are currently available for human, mouse, zebrafish, nematode, yeast, two bacterial species (*E. coli* K12 and *B. subtilis*) and two viral genomes (human cytomegalovirus and bacteriophage lambda).

## USAGE

**Figure 1.**
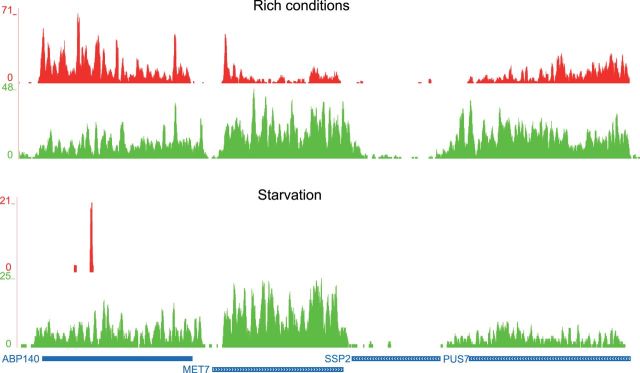
Observing differential translation in GWIPS-viz. Ribo-seq (red) and RNA-seq (green) coverage plots for the *S. cerevisiae* genome locus containing *ABP140*, *MET7*, *SSP2* and *PUS7* genes from (2). Under starvation conditions (bottom panel), *ABP140*, *MET7* and *PUS7* are transcribed but not translated.

In GWIPS-viz, users can search for their gene(s) of interest in the genome(s) for which ribo-seq data are available and view a snapshot of the gene’s translation under the conditions of the experiment. Ribosome coverage plots (red) and mRNA-seq coverage plots (green) display the number of reads that cover a given genomic coordinate. Figure 1 provides coverage plots for the *S. cerevisiae* genome locus containing *ABP140*, *MET7*, *SSP2* and *PUS7* from (2) and illustrates how differential translation can be viewed in GWIPS-viz.

**Figure 2.**
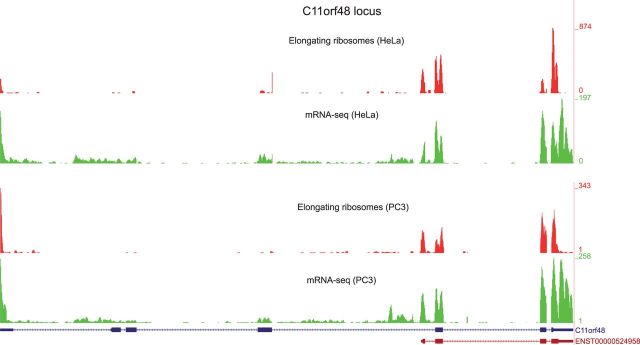
Comparing profiles from independent studies. Data from different studies and different organisms can be compared in GWIPS-viz. The C11orf48 locus in the human genome is shown where translation of an Ensembl transcript (brown bars) not annotated in RefSeq (blue bars) has been identified in HeLa cells (27). As can be seen, translation of the Ensembl transcript occurs in both HeLa (3) and PC3 human prostate cancer cells (6).

Users can visually identify which isoform(s) of a gene is transcribed and translated and also compare translation of the gene between different ribo-seq studies. For example, Figure 2 provides a comparison of two ribo-seq data sets obtained in different tissue-cultured human cells, HeLa (3) and PC3 human prostate cancer cells (6). It can be seen that translation of a non-Refseq Ensembl transcript, reported based on the analysis of HeLa cell data (27), is observed in both data sets.

**Figure 3.**
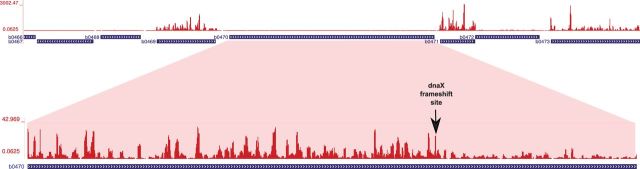
Ribo-seq data for the *dnaX* locus in the *E. coli* genome. The top panel corresponds to a segment containing neighbouring genes. The bottom panel contains the *dnaX* coordinates only. The displayed ribosome density is scaled relative to the maximum density within the selected region. The position of the programmed ribosomal frameshifting site in *dnaX* is indicated with an arrow.

For the eukaryotic data sets, ribosome profiles display the number of footprint reads at a particular genomic coordinate that align to the A-site (elongating ribosomes) or P-site (initiating ribosomes) of the ribosome, depending on the study. For the prokaryotic data sets, a weighted centred approach (18) is used to indicate the positions of ribosomes. Figure 3 shows ribosome profile densities in a region of the *E. coli* genome that includes the gene *dnaX* (b0470). The ribosome density is scaled relative to the maximum density present within the displayed genomic segment. As a result, in the zoomed segment allowing visualization of neighbour genes (top), *dnaX* appears as lowly expressed. However, at a range covering only the *dnaX* locus, it can be seen that nearly all codons in the *dnaX* mRNA are covered with footprints. Moreover the coverage is sufficient to allow visual detection of decreased ribosome density downstream of the site of programmed ribosomal frameshifting, which is known to cause ∼50% of translating ribosomes to terminate prematurely (28,29).

**Figure 4.**
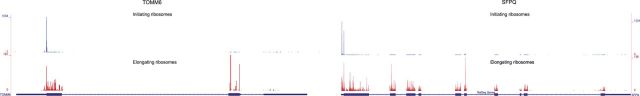
Combining profiles of initiating and elongating ribosomes. Profiles of initiating (blue) and elongating (red) ribosomes generated in human HEK 293 cells (7). Locations of elongating and initiating ribosomes are consistent with the annotated coding region of the *TOMM6* gene (left). However, ribosome profiles of the *SFPQ* gene point to the existence of an additional start codon (stronger peak) upstream of the annotated start codon (weaker peak).

Figure 4 provides an example of how ribo-seq tracks for elongating and initiating ribosomes can be compared. The example illustrates the data obtained in Human HEK293 cells (7) mapped to *TOMM6* and *SFPQ* genes. The latter gene apparently uses two sites of translation initiation for its expression.

## DATABASE DESIGN AND IMPLEMENTATION

GWIPS-viz is a customized version of the UCSC Genome Browser (26) version 269, and runs on Ubuntu Linux version 12.04.1, with Apache version 2.2.22 and MySQL 5.2.24. Static hypertext mark-up language and cascading style sheets files of the UCSC Genome Browser were downloaded from http://hgdownload.cse.ucsc.edu/and rehosted on our local server, whereas C source code for the common gateway interface executables was downloaded and compiled using gcc 4.6.3. Selected parts of the MySQL databases were downloaded from the UCSC browser for the majority of organisms included in GWIPS-viz.

Our partial mirror of the UCSC Genome Browser hosted on our server displays tracks for human (hg19), mouse (mm10), *S. cerevisiae* (sacCer3), zebrafish (danRer7), *C. elegans* (ce10), *E. coli* K12 (eschColi_K12), *B. subtilis* (baciSubt2), human cytomegalovirus (human herpesvirus 5 strain Merlin (HHV5)) and bacteriophage lambda (NC_001416) assemblies. Although several genome assemblies are available for many of the organisms, we chose to include only the most recent genome assembly for each organism.

Because the goal of GWIPS-viz is to be a browser for ribo-seq data, rather than a mirror of the UCSC browser, some of the functionality of the UCSC browser was removed to streamline the interface of GWIPS-viz. For example, the ‘clade’ menu in the genome selection menu was removed. In the browser window, the link ‘UCSC’ was added in the top bar to allow the user to view the current genome position in the UCSC browser.

Depending on the organism, certain tracks were retained from the UCSC browser (26) and were consolidated into one group called ‘Annotation Tracks’. Examples include RefSeq (30), Ensembl (31), Consensus Coding Sequence (32), conservation (33), RepeatMasker (Smit *et al.*, unpublished data, www.repeatmasker.org), mouse expressed sequence tags (34), Saccharomyces Genome Database genes (35) and transfer RNA genes (36).

Ribo-seq and mRNA-seq tracks were added by incorporating the outputs of our RNA-seq unified mapper (RUM) (37) alignment pipeline into the MySQL database. These tracks are divided into groups by publication and data type (ribo-seq and mRNA-seq). Tracks generated from uniquely mapping reads are colour coded according to their experiment type (elongating ribosome footprints are red, initiating ribosome footprints are blue and mRNA-seq reads are green).

### Raw sequencing data retrieval

Published Ribo-seq and mRNA-seq data sets are downloaded from the NCBI SRA (23) and converted to FASTQ format using the fastq-dump utility (SRA Handbook citation, not in PubMed). Data from replicate experiments are consolidated into one data set so as to have one browser track for each experimental condition.

### Alignment pipeline

As there are no specific tools as yet for aligning ribo-seq data, RNA-seq tools are used in our preprocessing and alignment pipeline.

Depending on the study, adaptor linker sequence or poly-(A) tails are trimmed from the 3′ ends of reads using Cutadapt version 1.1 (38). Trimmed reads shorter than 25 nt are discarded.

Contamination from ribosomal RNA (rRNA) may account for a significant proportion of the raw reads even after depletion by subtractive hybridization during the experiment. Hence it is desirable to remove rRNA reads from the data set before performing alignments to increase the proportion of informative sequences and improve alignment efficiency. To detect reads that are the result of rRNA contamination, trimmed reads are aligned to rRNA sequences using Bowtie (39). Bowtie version 0.12.8 is run using the -v option allowing three or fewer mismatches between the read sequence and the reference (rRNA) sequence. All reads that align to rRNA are discarded.

In most eukaryotes, a proportion of ribosome footprints will span splice junctions, i.e. the read will span the 3′ end of one exon and the 5′ end of another. There is the added complexity that ribo-seq reads are typically ∼30 nt in length. Hence the short-read alignment programme needs to be capable of aligning reads of ∼30 nt across splice junctions. We use the RUM, (current version 2.0.5_05) (37). RUM handles splice junctions by using the short read aligner Bowtie (39) to align sequence reads to both the genome and transcriptome and merging the results, before attempting to map remaining unaligned reads using another existing aligner, Blast-like alignment tool (40).

Owing to the relatively short lengths of ribosome footprint reads, a read may align to two or more distinct genomic locations due to sequence similarity. RUM outputs information separately for uniquely mapping reads and non-uniquely mapping reads (reads that align to several positions in the genome). Currently we provide tracks of uniquely mapping reads only in GWIPS-viz.

RUM’s output files include a Sequence Alignment/Map file showing the alignment(s) for each read, files giving the span of the alignment in genomic coordinates (RUM_Unique and RUM_NU) and coverage files (RUM.cov and RUM_NU.cov) listing the depth of coverage of reads across the genome.

The coverage files generated by the RUM alignment, RUM_Unique.cov and RUM_NU.cov, are in four column bedGraph format. The bedGraph data are converted into bigWig format, an indexed binary format that results in higher performance (41).

Ribosome profiles are generated from the RUM_Unique and RUM_NU files by obtaining the number of footprint reads whose 5′ ends align at a given genomic coordinate (with an offset of 12 nt designating the ribosome P-site for initiating ribosomes or 15 nt for the ribosome A-site for elongating ribosomes).

## FUTURE PLANS

We plan to expand the existing repertoire of ribo-seq tracks by integrating publically available ribosome profiling experiments as they become available.

GWIPS-viz currently displays the positions of the ribosomes mapped to the reference genomes. In the case of eukaryotic organisms that extensively use RNA splicing, visualization of ribosome positions in GWIPS-viz could be problematic due to a large number of long introns. Therefore, visualization of ribosome positions mapped to individual RNA transcripts is among our top priorities.

We currently provide ribo-seq and mRNA-seq tracks of uniquely mapping reads only. In the future, we wish to provide a differential display that will incorporate non-unique mapping reads (mapping to two or more locations in the genome) with uniquely mapping reads.

We also aim to provide access to the Galaxy platform from within GWIPS-viz so that researchers who generate their own ribo-seq experimental data can preprocess and align their data with the tools provided within Galaxy and then view the alignments in GWIPS-viz.

In addition, we aim to design a track specifically for the UCSC Genome Browser, which will display whether a region is translated or not (one global track per genome for which ribosome profiling data exist). If a user is interested in further details of the data (cell type or tissue, particular condition, specific density profile), they can be found in GWIPS-viz where individual tracks for each experiment are provided.

Our overall objective is to continuously improve the service we provide in GWIPS-viz. As GWIPS-viz is under intensive development, some of the features described in this article could become outdated soon. Hence we encourage users to post their questions, comments and feedback on the GWIPS-viz forum. Furthermore, as ribosome profiling is a relatively recent technique that is still evolving and undergoing optimization, we provide forums for discussing the experimental protocol itself, its applications and analysis of the data. In this way, GWIPS-viz will not only be a centralized repository to visualize ribosome profiling data, but its forums will encourage researchers to actively engage in the establishment of quality standards for ribosome profiling that will be of benefit to the community in general.

## FUNDING

Science Foundation Ireland [12/IA/1335 to P.V.B.]; Wellcome Trust [094423 to P.V.B.]. Funding for open access charge: The Wellcome Trust.

*Conflict of interest statement*. None declared.

## References

[1] Weiss RB, Atkins JF (2011). Molecular biology. Translation goes global. Science.

[2] Ingolia NT, Ghaemmaghami S, Newman JR, Weissman JS (2009). Genome-wide analysis *in vivo* of translation with nucleotide resolution using ribosome profiling. Science.

[3] Guo H, Ingolia NT, Weissman JS, Bartel DP (2010). Mammalian microRNAs predominantly act to decrease target mRNA levels. Nature.

[4] Stadler M, Fire A (2011). Wobble base-pairing slows in vivo translation elongation in metazoans. RNA.

[5] Reid DW, Nicchitta CV (2012). Primary role for endoplasmic reticulum-bound ribosomes in cellular translation identified by ribosome profiling. J. Biol. Chem..

[6] Hsieh AC, Liu Y, Edlind MP, Ingolia NT, Janes MR, Sher A, Shi EY, Stumpf CR, Christensen C, Bonham MJ (2012). The translational landscape of mTOR signalling steers cancer initiation and metastasis. Nature.

[7] Lee S, Liu B, Huang SX, Shen B, Qian SB (2012). Global mapping of translation initiation sites in mammalian cells at single-nucleotide resolution. Proc. Natl Acad. Sci. USA.

[8] Fritsch C, Herrmann A, Nothnagel M, Szafranski K, Huse K, Schumann F, Schreiber S, Platzer M, Krawczak M, Hampe J (2012). Genome-wide search for novel human uORFs and N-terminal protein extensions using ribosomal footprinting. Genome Res..

[9] Shalgi R, Hurt JA, Krykbaeva I, Taipale M, Lindquist S, Burge CB (2013). Widespread regulation of translation by elongation pausing in heat shock. Mol. Cell.

[10] Liu B, Han Y, Qian SB (2013). Cotranslational response to proteotoxic stress by elongation pausing of ribosomes. Mol. Cell.

[11] Loayza-Puch F, Drost J, Rooijers K, Lopes R, Elkon R, Agami R (2013). p53 induces transcriptional and translational programs to suppress cell proliferation and growth. Genome Biol..

[12] Ingolia NT, Lareau LF, Weissman JS (2011). Ribosome profiling of mouse embryonic stem cells reveals the complexity and dynamics of mammalian proteomes. Cell.

[13] Thoreen CC, Chantranupong L, Keys HR, Wang T, Gray NS, Sabatini DM (2012). A unifying model for mTORC1-mediated regulation of mRNA translation. Nature.

[14] Bazzini AA, Lee MT, Giraldez AJ (2012). Ribosome profiling shows that miR-430 reduces translation before causing mRNA decay in zebrafish. Science.

[15] Stadler M, Artiles K, Pak J, Fire A (2012). Contributions of mRNA abundance, ribosome loading, and post- or peri-translational effects to temporal repression of *C. elegans* heterochronic miRNA targets. Genome Res..

[16] Brar GA, Yassour M, Friedman N, Regev A, Ingolia NT, Weissman JS (2012). High-resolution view of the yeast meiotic program revealed by ribosome profiling. Science.

[17] Gerashchenko MV, Lobanov AV, Gladyshev VN (2012). Genome-wide ribosome profiling reveals complex translational regulation in response to oxidative stress. Proc. Natl Acad. Sci. USA.

[18] Oh E, Becker AH, Sandikci A, Huber D, Chaba R, Gloge F, Nichols RJ, Typas A, Gross CA, Kramer G (2011). Selective ribosome profiling reveals the cotranslational chaperone action of trigger factor *in vivo*. Cell.

[19] Li GW, Oh E, Weissman JS (2012). The anti-Shine-Dalgarno sequence drives translational pausing and codon choice in bacteria. Nature.

[20] Stern-Ginossar N, Weisburd B, Michalski A, Le VT, Hein MY, Huang SX, Ma M, Shen B, Qian SB, Hengel H (2012). Decoding human cytomegalovirus. Science.

[21] Liu X, Jiang H, Gu Z, Roberts JW (2013). High-resolution view of bacteriophage lambda gene expression by ribosome profiling. Proc. Natl Acad. Sci. USA.

[22] Michel AM, Baranov PV (2013). Ribosome profiling: a Hi-Def monitor for protein synthesis at the genome-wide scale. Wiley Interdiscip. Rev. RNA.

[23] Shumway M, Cochrane G, Sugawara H (2010). Archiving next generation sequencing data. Nucleic Acids Res..

[24] Nekrutenko A, Taylor J (2012). Next-generation sequencing data interpretation: enhancing reproducibility and accessibility. Nat. Rev. Genet..

[25] Blankenberg D, Von Kuster G, Coraor N, Ananda G, Lazarus R, Mangan M, Nekrutenko A, Taylor J (2010). Galaxy: a web-based genome analysis tool for experimentalists. Curr. Protoc. Mol. Biol..

[26] Meyer LR, Zweig AS, Hinrichs AS, Karolchik D, Kuhn RM, Wong M, Sloan CA, Rosenbloom KR, Roe G, Rhead B (2013). The UCSC Genome Browser database: extensions and updates 2013. Nucleic Acids Res..

[27] Michel AM, Choudhury KR, Firth AE, Ingolia NT, Atkins JF, Baranov PV (2012). Observation of dually decoded regions of the human genome using ribosome profiling data. Genome Res..

[28] Larsen B, Gesteland RF, Atkins JF (1997). Structural probing and mutagenic analysis of the stem-loop required for *Escherichia coli* dnaX ribosomal frameshifting: programmed efficiency of 50%. J. Mol. Biol..

[29] Tsuchihashi Z, Kornberg A (1990). Translational frameshifting generates the gamma subunit of DNA polymerase III holoenzyme. Proc. Natl Acad. Sci. USA.

[30] Pruitt KD, Tatusova T, Brown GR, Maglott DR (2012). NCBI Reference Sequences (RefSeq): current status, new features and genome annotation policy. Nucleic Acids Res..

[31] Flicek P, Ahmed I, Amode MR, Barrell D, Beal K, Brent S, Carvalho-Silva D, Clapham P, Coates G, Fairley S (2013). Ensembl 2013. Nucleic Acids Res..

[32] Harte RA, Farrell CM, Loveland JE, Suner MM, Wilming L, Aken B, Barrell D, Frankish A, Wallin C, Searle S (2012). Tracking and coordinating an international curation effort for the CCDS Project. Database (Oxford).

[33] Pollard KS, Hubisz MJ, Rosenbloom KR, Siepel A (2010). Detection of nonneutral substitution rates on mammalian phylogenies. Genome Res..

[34] Benson DA, Karsch-Mizrachi I, Lipman DJ, Ostell J, Wheeler DL (2004). GenBank: update. Nucleic Acids Res..

[35] Cherry JM, Hong EL, Amundsen C, Balakrishnan R, Binkley G, Chan ET, Christie KR, Costanzo MC, Dwight SS, Engel SR (2012). *Saccharomyces* Genome Database: the genomics resource of budding yeast. Nucleic Acids Res..

[36] Chan PP, Lowe TM (2009). GtRNAdb: a database of transfer RNA genes detected in genomic sequence. Nucleic Acids Res..

[37] Grant GR, Farkas MH, Pizarro AD, Lahens NF, Schug J, Brunk BP, Stoeckert CJ, Hogenesch JB, Pierce EA (2011). Comparative analysis of RNA-Seq alignment algorithms and the RNA-Seq unified mapper (RUM). Bioinformatics.

[38] Martin M (2011). Cutadapt removes adapter sequences from high-throughput sequencing reads. EMBnet J..

[39] Langmead B, Trapnell C, Pop M, Salzberg SL (2009). Ultrafast and memory-efficient alignment of short DNA sequences to the human genome. Genome Biol..

[40] Kent WJ (2002). BLAT–the BLAST-like alignment tool. Genome Res..

[41] Kent WJ, Zweig AS, Barber G, Hinrichs AS, Karolchik D (2010). BigWig and BigBed: enabling browsing of large distributed datasets. Bioinformatics.

